# 1D solitons in cubic-quintic fractional nonlinear Schrödinger model

**DOI:** 10.1038/s41598-022-19332-z

**Published:** 2022-09-02

**Authors:** V. A. Stephanovich, W. Olchawa, E. V. Kirichenko, V. K. Dugaev

**Affiliations:** 1 Institute of Physics, University of Opole ul., Oleska 48, 45-052 Opole, Poland; 2 Department of Physics and Medical Engineering, Rzeszów University of Technology, al. Powstańców Warszawy 6, 35-959 Rzeszów, Poland

**Keywords:** Solitons, Nonlinear phenomena, Quantum mechanics

## Abstract

We examine the properties of a soliton solution of the fractional Schrö dinger equation with cubic-quintic nonlinearity. Using analytical (variational) and numerical arguments, we have shown that the substitution of the ordinary Laplacian in the Schrödinger equation by its fractional counterpart with Lévy index $\alpha$ permits to stabilize the soliton texture in the wide range of its parameters (nonlinearity coefficients and $\alpha$) values. Our studies of $\omega (N)$ dependence ($\omega$ is soliton frequency and *N* its norm) permit to acquire the regions of existence and stability of the fractional soliton solution. For that we use famous Vakhitov-Kolokolov (VK) criterion. The variational results are confirmed by numerical solution of a one-dimensional cubic-quintic nonlinear Schrödinger equation. Direct numerical simulations of the linear stability problem of soliton texture gives the same soliton stability boundary as within variational method. Thus we confirm that simple variational approach combined with VK criterion gives reliable information about soliton structure and stability in our model. Our results may be relevant to both optical solitons and Bose-Einstein condensates in cold atomic gases.

## Introduction

Nonlinear phenomena are realized in many branches of physics ranging from nonlinear optics (like self-focusing of laser beams), and Bose-Einstein condensation (BEC), to the theory of elasticity^1–6^. In this case, the nonlinear Schrödinger equation (NLSE) is utilized for their description. This equation expresses a balance between the dispersion (kinetic energy term, reduced to the Laplacian in ordinary systems) and nonlinearity and had been successfully applied to different nonlinear systems. To name o few, these are the systems with power-law (like cubic, quintic, and mixed cubic-quintic) and saturable nonlinearities^2–8^. In nonlinear optics, the NLSE is a basic tool to investigate light propagation in nonlinear optical media^1,4,5^. The majority of light beam shapes in this case are solitons - the nonlinear solitary waves, emerging as a result of a balance of nonlinearity and dispersion^2,9^. Because of their ability to propagate without altering their shapes, optical solitons have a promising future as primary signal carriers in telecommunication. It has been demonstrated in recent review articles^6,10^, that if the dispersion has the exotic ”fractional” form, the picture becomes richer and much more interesting physically, than in an ordinary case. Below we will also demonstrate this feature. One more example of the NLSE with cubic nonlinearity is the Gross-Pitaevskii equation (GPE), which is extensively used to study the mean field properties of the ground state of a system of identical bosons and thus is a central equation for BEC in ultracold bosonic gases^3^. In this case, the solitons with fractional dispersion play an important role, especially in the presence of spin-orbit coupling^11^. In this context, we also note the review paper^6^, where large corps of recent theoretical and experimental results dedicated to nonlinear localized textures in diverse media (ranging from systems with BEC to various optical setups) has been reviewed. This also includes the systems with fractional dispersion.

The description of boson systems in terms of GPE is appropriate only at a sufficiently low density of condensate, where two-body interactions dominate^3^. For higher densities three-body interactions enter the scene, generating quintic nonlinear terms in the corresponding NLSE. Thus, at higher boson density, the BECs are described by the NLSE with mixed cubic-quintic nonlinearity^12,13^. The one-dimensional (1D) NLSE with mixed cubic-quintic nonlinearity appears also in the context of optical pulses propagation in double-doped optical fibers with nonlinear effective refraction index^1,14^.

In dimensionless units ($\hbar =m=1$, where *m* is the mass of a boson in BEC setup) this equation has the form

1 $$\begin{aligned} i\frac{\partial \psi }{\partial t}=-\frac{\partial ^2 \psi }{\partial x^2}+g|\psi |^2\psi +\chi |\psi |^4\psi , \end{aligned}$$

where *g* and $\chi$ are the nonlinear coefficients, corresponding to the two- (cubic nonlinearity) and three-body (quintic nonlinearity) interactions respectively. The signs of *g* and $\chi$ could be positive (repulsive interaction) or negative (attractive interaction) respectively. The substitution

2 $$\begin{aligned} \psi (x,t)=y(x)e^{i\omega t}, \end{aligned}$$

($\omega$ is a soliton frequency) recasts (1) into the following form

3 $$\begin{aligned} \frac{d^2y}{dx^2}-\omega y - gy^3 - \chi y^5=0, \end{aligned}$$

The soliton solutions of the equation (3) had been found in many papers (see, e.g.^15–17^) for different sign combinations of *g* and $\chi$. It can be shown that these solutions, especially for the case of attractive interaction $g, \chi <0$ are unstable according to Vakhitov-Kolokolov (VK) criterion^18^, being prone to either collapse or decay to zero. There exist several methods to stabilize such soliton texture. One of them is to use so-called optical lattice (a spatially periodic polarization pattern, formed by counter-propagating laser beams) or external parabolic potential^7,8^. The other one is dynamic stabilization, where the time dependence is assigned to the coefficients *g* and $\chi$. This is called nonlinearity management and is accomplished in such a way that the resulting soliton texture should avoid collapse (so-called collapse arrest) and otherwise become stable^19^.

Here we suggest one more method of soliton texture stabilization. It is related to the random management of the above NLSE in terms of so-called fractional derivatives, which describe non-Gaussian probability distributions. The former derivatives, in particular, generate so-called Lévy stable distributions^20–23^. The peculiar feature of these distributions is the power-law $|x|^{-1-\alpha }$ decay of their probability densities, where $0<\alpha < 2$ is the Lévy index. The latter character of decay means that Lévy stable distributions decrease much slower than ordinary Gaussian at $\alpha \ne 2$. It can be shown that the case $\alpha =2$ corresponds to the conventional Gaussian distribution. To be specific, below we will show, that the replacement of ordinary Laplacian (second spatial derivative in 1D case) by fractional one in the NLSE (1) stabilizes its soliton solutions.

The Schrödinger equation with ordinary Laplacian being substituted by fractional one had been introduced by Laskin in the context of fractional quantum mechanics^24^. The introduction of the fractional Schrödinger equation was made possible in the Feynman picture of quantum mechanics^25^ , based on the path integral over all possible trajectories of a quantum particle. Namely, in fractional quantum mechanics, the path integral is taken over the trajectories, obeying Lévy (instead of Gaussian) statistics. In this case, as Lévy index $\alpha$ is responsible for the deviation of the underlying system trajectories from Gaussian ones, this quantity plays a role of a phenomenological descriptor of the degree of disorder.

The fractional NLSE with cubic-quintic nonlinearities had been widely studied, see^26,27^ and references therein. In the present paper, using analytical (variational) and numerical arguments, we study the structure of soliton solutions of the above equation. As an introduction of the fractional derivatives in NLSE may be related to the disorder, we may consider the substitution of the ordinary Laplacian to the fractional one as a kind of disorder management or disorder engineering, leading to the soliton textures stabilization. So, one more aim of the present study is to address the possibility of soliton stabilization by the above disorder management, reduced in our case to the introduction of the fractional derivatives in the corresponding NLSE.

Our variational and numerical results reveal the ”phase diagram” of the soliton existence and stability in terms of variables $\lambda =\sqrt{3}g/(4\sqrt{\omega \chi })$ (combination of parameters, entering the soliton solution of Eq. (3), see below) - $\alpha$ (Lévy index). For $g=0$, (corresponding to $\lambda =0$) we obtain our previous result^28^ that stable soliton textures exist in fractional NLSE with quintic nonlinearity at $2/3<\alpha <2$.

The plan of our paper is as follows. The basic fractional NLSE with cubic-quintic nonlinearity is discussed in the following section “The model”. Then, in the section ”Analytical results: the variational approach”, we present the variational method and obtain the soliton structure. The same method permits to investigate soliton stability on the base of VK criterion. This, in turn, allows to outline the existence and stability domains for the fractional solitons. Latter results, based on the computation of eigenvalues for small perturbations, are confirmed by direct numerical simulations of perturbed dynamics of the solitons in the section “Numerical results: soliton stability”. The last section "Conclusions" is devoted to possible generalizations of the model considered as well as to the physical implications of the results obtained.

## The model

We consider the substitution of the ordinary second derivative in the NLSE (1) by the 1D fractional Laplacian, which is Riesz fractional derivative, defined by the following integral relation^22–24,29,30^

4 $$\begin{aligned} |\Delta |^{\alpha /2}g(x)= & {} A_\alpha \int _{-\infty }^{\infty }\frac{g(u+x)-g(x)}{|u|^{1+\alpha }}du, \end{aligned}$$

5 $$\begin{aligned} A_\alpha= & {} \frac{\Gamma (1+\alpha )}{\pi }\sin \frac{\pi \alpha }{2}, \end{aligned}$$

where $0<\alpha <2$ is the Lévy index. Note that at $\alpha =2$ the fractional 1D Laplacian (5) converts into ordinary second spatial derivative. The details of the corresponding transition are listed in the Appendix.

As the integral in (4) exists as the Cauchy principal value only, it is convenient to represent it in the form of the Fourier image, see Appendix for details

6 $$\begin{aligned} |\Delta |^{\alpha /2}f(x)=-\frac{1}{2\pi }\int _{-\infty }^\infty |k|^\alpha f(k)e^{-ikx}dk. \end{aligned}$$

The expression (6) implies that the Fourier image of the fractional Laplacian is simply $-|k|^\alpha$, which for $\alpha =2$ yields the usual second derivative. The fractional equation, having soliton solutions for $g, \chi <0$, is obtained by substitution of (2) into (1) with respect to (4), (5). This generates following explicit equation for *y*(*x*)

7 $$\begin{aligned} A_\alpha \int _{-\infty }^{\infty }\frac{y(p+x)-y(x)}{|p|^{1+\alpha }}dp-\omega y +|g| y^3+|\chi | y^5=0, \end{aligned}$$

where $A_\alpha$ is defined by (5). At $\alpha =2$ the equation (7) transforms into (3), which for this case has the exact soliton solution^15–17^ $y_{\alpha =2}\equiv y_0$

8 $$\begin{aligned} y_0= & {} \frac{A_0}{\sqrt{\lambda +\sqrt{1+\lambda ^2}\cosh B_0x}},\ A_0=\left( \frac{3\omega }{\chi }\right) ^{1/4},\nonumber \\ B_0= & {} 2\sqrt{\omega },\ \lambda =\frac{\sqrt{3}g}{4\sqrt{\omega \chi }}\equiv \sqrt{\frac{\omega _0}{\omega }},\ \omega _0=\frac{3g^2}{16\chi }. \end{aligned}$$

The parameters $\lambda$ and $\omega _0$ will be necessary in subsequent calculations. Also, in (8), we assume $g,\chi >0$ so that we omit moduli signs. The solution (8) gives the interpolation between the cases of pure cubic ($\chi =0$ corresponding to $\lambda \rightarrow \infty$) and quintic ($g=0$, corresponding to $\lambda =0$) nonlinearities.

The norm of the solution (8) reads

9 $$\begin{aligned} N_0=\int _{-\infty }^\infty y_0^2dx=\sqrt{\frac{3}{\chi }}\left[ \frac{\pi }{2}-\arctan \lambda \right] . \end{aligned}$$

First, we see that it also interpolates between known cases of quintic

10 $$\begin{aligned} N_0(\lambda =0)=\frac{\pi }{2}\sqrt{\frac{3}{\chi }} \end{aligned}$$

and cubic

11 $$\begin{aligned} N_0(\lambda \rightarrow \infty )=\frac{4}{g}\sqrt{\omega } \end{aligned}$$

nonlinearities. Second, we see that for both asymptotic solutions the famous VK stability criterion^18^ $dN/d\omega <0$ does not hold. Namely, for the solution with quintic nonlinearity (10) we have $dN/d\omega =0$, i.e. so-called marginal stability^7,8^. At the same time, the solution with cubic nonlinearity (11) is plainly unstable, i.e. $dN/d\omega >0$ for it. In the mixed case (9) we have

12 $$\begin{aligned} \frac{dN_0}{d\omega }=\frac{3}{8\chi }\frac{1}{\omega ^{3/2}(1+\lambda ^2)}>0, \end{aligned}$$

which also means soliton instability. Below we show that the introduction of the fractional derivatives will stabilize the resulting soliton texture for $\alpha <2$. Also, as for $\alpha =2$, the mixed ”cubic-quintic soliton” is unstable, in the fractional case (7) of arbitrary $\alpha <2$ we will have the entire ”phase diagram” of soliton existence and stability in terms of variables $\alpha$ - $\lambda$.

## Analytical results: the variational approach

The variational functional corresponding to the nonlinear equation (7), has the form

13 $$\begin{aligned} W_\alpha =\int _{-\infty }^\infty dx\bigg [-\frac{1}{2} \left( |\nabla |^{\alpha /2}y\right) ^2-\omega \frac{y^2}{2}\nonumber \\ +g\frac{y^4}{4}+\chi \frac{y^6}{6}\bigg ],\ g,\chi >0. \end{aligned}$$

Here the first term is the fractional gradient, which is best defined via its Fourier transform

14 $$\begin{aligned} |\nabla |^{\alpha /2}y(x)=-\frac{1}{2\pi }\int _{-\infty }^\infty |k|^{\alpha /2} y(k)e^{-ikx}dk. \end{aligned}$$

Note that at $\alpha =2$, the first term in (13) gives the square of the ordinary first spatial derivative.

The next step is to substitute the trial function, defining the soliton texture, into the energy function (13). To keep things as simple as possible, it is reasonable to consider the ”ordinary” solution (8) as a trial function. In this case we substitute the fixed parameters $A_0$ and $B_0$ with variational ones. Specifically, we consider the trial function of the form

15 $$\begin{aligned} y(x)=Af(Bx),\ f(z)=\frac{1}{\sqrt{\lambda +\sqrt{1+\lambda ^2}\cosh z}}, \end{aligned}$$

where *A* and *B* are variational parameters. The best way to calculate the integral of the fractional gradient in the energy (13) for the trial function (15) is to use the Fourier image *y*(*k*) of the function *y*(*x*). Namely,

16 $$\begin{aligned} y(k)= & {} A\int _{-\infty }^\infty f(Bx)e^{ikx}dx\nonumber \\= & {} \frac{A}{B}\int _{-\infty }^\infty f(z)e^{i\frac{k}{B}z}dz\equiv \frac{A}{B}{\tilde{y}}\left( \frac{k}{B}\right) . \end{aligned}$$

Substitution of the inverse Fourier image (14) of the function (16) into the integrand of the first term in (13) generates factor $e^{-ix(k+k')}$ in it. After integration over *x* this yields $2\pi \delta (k+k')$, where $\delta (z)$ is Dirac $\delta$ function. After integration over $k'$ we than arrive at the following expression for the first term. Substitution of (14) with respect to (16) into the first term (13) yields

17 $$\begin{aligned} I_{1\alpha }= & {} -\frac{1}{2} \int _{-\infty }^\infty \left( |\nabla |^{\alpha /2}y\right) ^2 dx= -\frac{1}{8\pi ^2}\int _{-\infty }^\infty |k|^{\alpha /2} \nonumber \\&\quad \times |k'|^{\alpha /2} y(k) y(k')dk dk' \int _{-\infty }^\infty e^{-i(k+k')x}dx \nonumber \\= & {} -\frac{1}{4\pi }\int _{-\infty }^\infty |k|^{\alpha /2} |k'|^{\alpha /2} y(k) y(k')\delta (k+k')dk dk' \nonumber \\= & {} -\frac{1}{4\pi } \int _{-\infty }^\infty |k|^\alpha y^2(k)dk \nonumber \\= & {} -\frac{1}{2\pi }\frac{A^2}{B^2}\int _0^\infty k^\alpha {{\tilde{y}}}^2\left( \frac{k}{B}\right) dk \equiv -\kappa _\alpha A^2B^{\alpha -1}, \nonumber \\ \kappa _\alpha= & {} \frac{1}{2\pi }\int _0^\infty z^\alpha {{\tilde{y}}}^2(z)dz. \end{aligned}$$

Here we used the fact that Fourier image (16) is the even function of its argument. The variational energy then yields

18 $$\begin{aligned} E_\alpha =-\kappa _\alpha A^2B^{\alpha -1}+\frac{\langle f^6 \rangle }{B}\bigg [\chi \frac{A^6}{6}+ g \frac{\langle f^4 \rangle }{\langle f^6 \rangle }\frac{A^4}{4}-\frac{\omega }{2} A^2\frac{\langle f^2 \rangle }{\langle f^6 \rangle }\bigg ], \end{aligned}$$

where $\langle f^n \rangle =\int _{-\infty }^\infty f^n(x)dx$. The functions $\langle f^n \rangle$ ($n=2,4,6$) have following explicit form

19 $$\begin{aligned} \langle f^2 \rangle= & {} 2\left[ \frac{\pi }{2}-\arctan \lambda \right] , \nonumber \\ \langle f^4 \rangle= & {} 2\left[ 1-\lambda \left( \frac{\pi }{2}-\arctan \lambda \right) \right] , \nonumber \\ \langle f^6 \rangle= & {} (1+3\lambda ^2)\left( \frac{\pi }{2}-\arctan \lambda \right) -3\lambda . \end{aligned}$$

**Figure 1:**
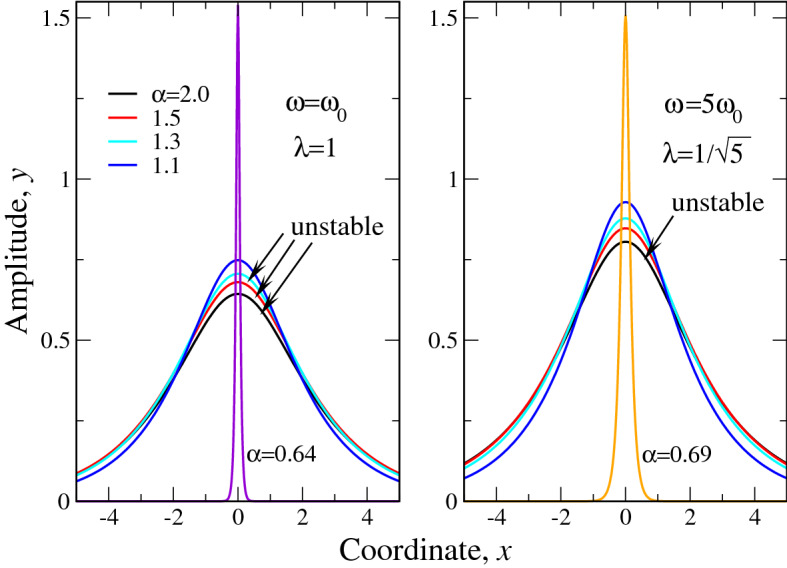
The variational solution (21) for the fractional soliton for $\omega =\omega _0$ ($\lambda =1$, left panel) and $\omega =5\omega _0$ ($\lambda =1/\sqrt{5}$, right panel), plotted for Lévy indices $\alpha$ (legend in the left panel) down to their critical values $\alpha _{cr}(\lambda )$ for soliton existence. Namely, for $\omega =\omega _0$ ($\lambda =1$) $\alpha _{cr} \approx 0.6266$, while for $\omega =5\omega _0$ ($\lambda =1/\sqrt{5}$) $\alpha _{cr} \approx 0.6491$. We plot the critical soliton textures for $\alpha$ (shown near the corresponding curves), slightly larger that the critical values, for better visualization. Curves, labeled ”unstable”, correspond to Lévy indices, for which $dN/d\omega >0$, i.e. VK stability criterion does not fulfilled.

It is seen from (19) that functions $\langle f^n \rangle$ are all positive and independent of *A* and *B*. This means that we can find the extremum of $E_\alpha$ (18) with respect to these parameters. After lengthy calculations we arrive at following result

20 $$\begin{aligned}&a^2=\frac{2}{3} \beta (\lambda ) \frac{\alpha - \frac{1}{2}}{\alpha - \frac{2}{3}}\left[ -1+\sqrt{1+\gamma (\lambda )\frac{\alpha \left( \alpha -\frac{2}{3}\right) }{\left( \alpha - \frac{1}{2}\right) ^2}}\right] , \nonumber \\&b^\alpha = \frac{\langle f^2\rangle -\lambda \langle f^4\rangle a^2}{4(3\mu -2)\kappa _\alpha },\ a\equiv \frac{A}{A_0}, \ b\equiv \frac{B}{B_0}, \nonumber \\&\beta (\lambda )=\lambda \frac{\langle f^4 \rangle }{\langle f^6 \rangle },\ \gamma (\lambda )=\frac{3}{4\lambda ^2}\frac{{\langle f^2 \rangle }{\langle f^6 \rangle }}{{\langle f^4 \rangle }^2}, \end{aligned}$$

where parameters $A_0$, $B_0$ are defined by Eq. (8) and $\kappa _\alpha$ by (17). The requirement of positivity of the above parameters $a^2$ and $b^\alpha$, generates the phase diagram of the soliton solution existence in terms of variables $\alpha$ - $\lambda$. This phase diagram will be considered below with respect also to the line $dN/d\omega =0$, which defines the borders of the soliton stability. Our explicit numerical solution of the fractional equation (7) along with simulations of the soliton texture stability will show that the above border, obtained variationally, describes the numerical one with the very good accuracy, not exceeding 1%.

Note, that in Refs.^10,11^, the variational approach has also been applied to study the soliton textures in the systems with fractional Laplacians. Thus, it is instructive to compare our variational approach with those devised in the above references. Namely, in Ref.^11^, the variational method has been used to study the 1D and 2D solitons in Gross-Pitaevsky equations with spin-orbit interaction. In this case, the solution is a two-component spinor and thus is more complicated than our trial function (15). Also, as the exact solution of the problem with ordinary (i.e. that at $\alpha =2$) Laplacian is unknown, the authors^11^ use the general Gaussian variational *ansätse* for both spinor components. At the same time, the 2D ”mathematical counterpart” of our system has also been studied in Ref.^10^. Formally, it comprises one component two-dimensional nonlinear system with cubic-quintic nonlinearity and fractional kinetic energy (Laplacian). This corresponds physically to the vortex soliton textures in systems with fractional diffraction. Here, the numerical (instead of variational) solution of the corresponding fractional equation has been used to extract the information about the soliton structure. The 2D variational solutions have been provided for other solitons types like those in trapping potentials.

This shows the multitude of possibilities for relatively simple variational approaches in the investigation of solitons structure, especially in higher dimensions, where the direct numerical simulations become quite computer-intensive and require additional care as the numerical scheme by itself may become unstable.

Substitution of the energy minimizing parameters (20) into the trial function (15) generates following variational soliton solution

21 $$\begin{aligned} y_1=af(bx_1),\ y_1=\frac{y}{A_0},\ x_1=B_0x. \end{aligned}$$

Here, the function *f*(*z*) is defined by (15). The variational solution (21) is reported in Fig. 1 for two selected values of soliton frequency in the units of $\omega _0$ (8). The shape of solutions at other $\omega$ is qualitatively similar to those shown in Fig. 1. It is seen that as Lévy index approaches the corresponding critical values ($\alpha _{cr} \approx 0.6266$ for $\omega =\omega _0$ and $\alpha _{cr} \approx 0.6491$ for $\omega =5\omega _0$), the solution becomes progressively more peaked so that at $\alpha \rightarrow \alpha _{cr}$ the peak height goes to infinity. That being said, at $\alpha \rightarrow \alpha _{cr} (\lambda )$ we have the soliton collapse due to “excessive fractionality”. Note, that as Lévy index can be viewed as a measure of disorder in a system (see, e.g.^22,28,31–33^), the values $\alpha _{cr}$ can be regarded as some threshold disorder strength, at which the solitons cease to exist in a system.

**Figure 2:**
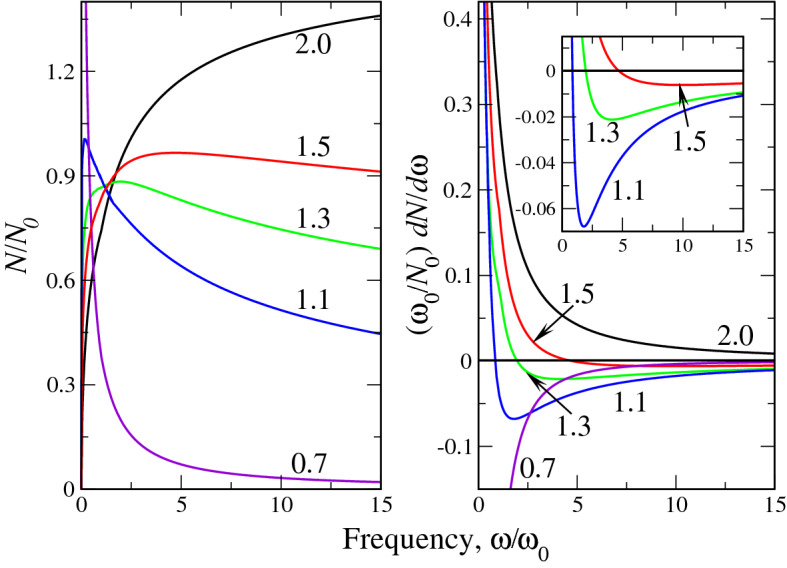
The dimensionless fractional soliton norm, calculated variationally (left panel) and its frequency derivative (right panel). Figures near curves in both panels correspond to the Lévy indices $\alpha$. Inset in right panel shows the negative parts of $dN/d\omega$ for $1.1<\alpha <1.5$ in larger scale.

For the variational solution (21), the soliton norm can be easily expressed in the form

22 $$\begin{aligned} N=\frac{A^2}{B} \langle f^2 \rangle , \end{aligned}$$

which permits to obtain the explicit expression for $N(\omega )$ in fractional case. Substitution of (19) and (20) into (22) yields

23 $$\begin{aligned} N(\omega )= & {} N_0\Omega ^{\frac{1}{2}-\frac{1}{\alpha }}\frac{a^2(\Omega )}{b(\Omega )}\left[ \frac{\pi }{2}-\arctan \frac{1}{\sqrt{\Omega }}\right] , \nonumber \\ N_0= & {} \frac{2\sqrt{3}}{4^{\frac{1}{\alpha }}\sqrt{\chi }}\omega _0^{\frac{1}{2}-\frac{1}{\alpha }}, \ \Omega =\frac{\omega }{\omega _0}. \end{aligned}$$

Here *a* and *b* are minimizing variational parameters (20). They are the functions of parameter $\lambda$ and by this virtue of $\Omega$, see Eq. (8). It is seen that at $\alpha =2$, the dependence $N(\omega )$ (23), identically gives (9), derived for the ”ordinary” soliton. The dependence $N(\omega )$ (23) as well as its frequency derivative is shown in Fig. 2. It is seen from the left panel that at $\alpha =2$ the dependence $N(\omega )$ increases monotonically so that $dN/d\omega >0$ everywhere. According to VK criterion^18^ this means that the soliton texture is unstable. On the other hand, our analysis of the expression (23) shows, that as soon as $\alpha$ becomes less than 2 (fractional case), the dependence $N(\omega )$ stops being monotonously growing. Rather, it acquires a maximum, where $dN/d\omega =0$ and then decreases, giving rise to stable soliton textures with $dN/d\omega <0$. More specifically, an infinitesimal deviation of $\alpha$ from 2 generates such a maximum at very large $\Omega \rightarrow \infty$. At smaller $\alpha$ this maximum shifts towards smaller, finite $\omega /\omega _0$ (see, e.g., the curves $N(\omega )$ on the left panel of Fig. 2, corresponding to $\alpha =1.5$ and 1.3) and finally, at $\alpha \rightarrow 1$, it occurs at $\omega =0$ and at $\alpha < 1$ disappears. This gives rise to complete soliton stability at $\alpha _{cr}<\alpha <1$, where $\alpha _{cr}$ depends on $\Omega$, see Fig. 1. The collection of points $\Omega$, at which $dN/d\omega =0$ comprises the curve, where the stable soliton starts to exist. This curve will be shown below, in the soliton phase diagram. The right panel of Fig. 2 visualizes the above behavior in terms of the derivative $dN/d\omega$ (specifically, $d(N/N_0)/d\Omega$). It is seen that while the derivative is strictly positive at $\alpha =2$, at $\alpha =1.5$ there is already the negative part of the $dN/d\omega$ curve (see also inset, which shows this in more details) and at $\alpha =0.7$ the derivative is strictly negative, going to very large negative numbers as $\omega \rightarrow 0$.

**Figure 3:**
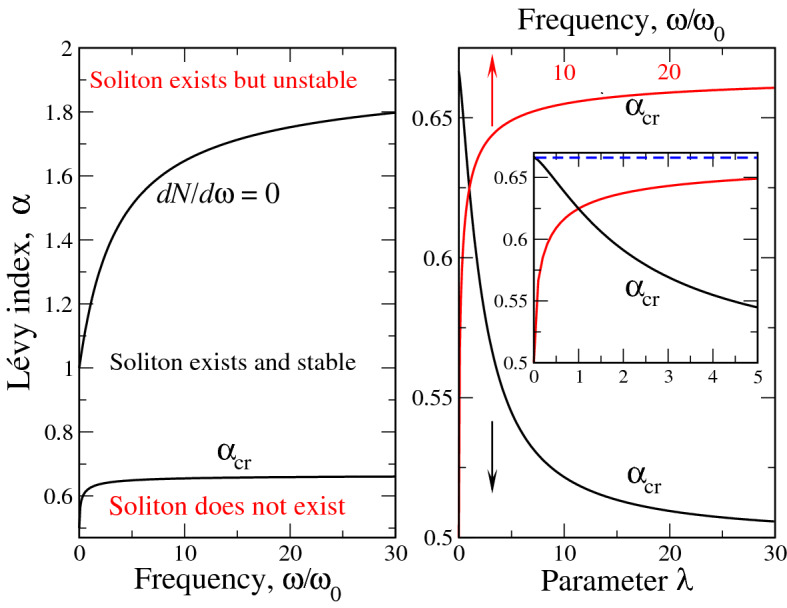
The ”phase diagram” of the soliton under consideration. Left panel shows the boundaries of soliton existence ($1/2<\alpha _{cr}<2/3$) and stability $dN/d\omega =0$ versus dimensionless frequency $\omega /\omega _0$. The intervals of soliton existence and stability are shown in the panel. Right panel reports the soliton existence boundary $\alpha _{cr}$ as a function of parameters $\lambda = (\omega /\omega _0)^{-1/2}$ (black curve) or $\omega /\omega _0$ (red curve). Inset in right panel reports the details of $\alpha _{cr}(\omega /\omega _0)$ at small $\omega$. Dashed line in the inset corresponds to the $g \rightarrow 0$ (or $\omega _0 \rightarrow 0$) asymptotics ($\alpha _{cr}=2/3$, corresponding to the case of pure quintic nonlinearity) of soliton existence.

The soliton ”phase diagram” is reported on the left panel of Fig. 3 in terms of variables $\alpha$ - $\Omega$. The right panel of Fig. 3 shows that it can be represented equally well in terms of $\lambda =1/\sqrt{\Omega }$ rather than $\Omega$. However, it seems to us to be ”more physical” to use variable $\Omega =\omega /\omega _0$ as it explicitly relates the actual soliton frequency to the combination of cubic *g* and quintic $\chi$ nonlinearity coefficients. It is seen from the left panel, that the stable soliton textures exist between two curves: upper $dN/d\omega =0$ (stability boundary) and lower defining $\alpha _{cr}(\Omega )$, where the soliton transforms to something similar to Dirac $\delta$ - function. Right panel of Fig. 3 shows that the latter curve interpolates between the cases of pure quintic nonlinearity ($g=0$) with $\alpha _{cr}=2/3$ and pure cubic nonlinearity ($\chi =0$) having $\alpha _{cr}=1/2$. As the case of quintic nonlinearity ($g=0$) corresponds to $\omega _0 \rightarrow 0$ (see Eq. (8)), at large $\Omega$ soliton begins to stabilize ($N(\omega )$ acquires maximum) already for $\alpha$ infinitesimally less than 2, see the discussion above. This is because in the case of quintic nonlinearity ($g=\omega _0=0$) the soliton texture stabilizes immediately as the equation ”fractionalizes”, i.e. $\alpha$ becomes less than 2, see Ref.^28^ for details. At the same time, in the opposite limiting case of cubic nonlinearity ($\chi =0$, $\omega _0 \rightarrow \infty$, $\Omega \rightarrow 0$), the stable soliton exists at $\alpha <1$ only. This explains the transition of the curve $dN/d\omega =0$ from $\Omega \rightarrow \infty$ at $\alpha \rightarrow 2$ to $\Omega \rightarrow 0$ at $\alpha =1$. The Fig. 3 explains why some curves in Fig. 1 are labeled ”unstable”. This is because their Lévy indices $\alpha$ lie above the stability boundary $dN/d\omega =0$ for $\omega /\omega _0=1$ and 5 respectively.

This shows that above simple variational approach not only permits to obtain the fractional soliton structure, but predicts its stability (within VK criterion) at certain range of Lévy indices and dimensionless soliton frequencies $\Omega$, which includes nonlinearity coefficients *g* and $\chi$. This is one of the main results of the present consideration, which will be confirmed below by direct numerical simulations.

## Numerical results: soliton stability

**Figure 4:**
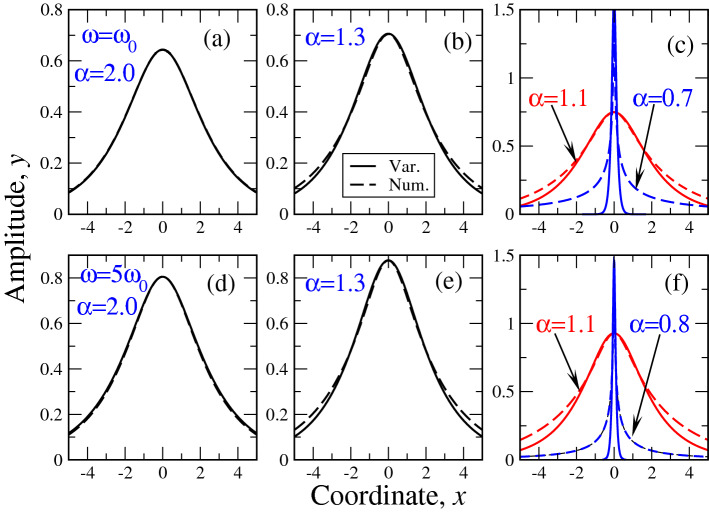
Comparison of numerical (dashed lines) and variational (solid lines) solutions of the fractional soliton equation (7) for different frequencies (in the units of $\omega _0$, see legends in (a) and (d)) and Lévy indices $\alpha$, shown in the legends. Upper row (panels (a),(b),(c)) corresponds to $\omega =\omega _0$, lower (panels (d),(e),(f)) - to $\omega =5\omega _0$.

To check the accuracy of the above variational approach, it is instructive to compare its results with the direct numerical solution of the equation (7). For that, we bring this equation to the units (21). The comparison of numerical and variational results for selected values of $\alpha$ at both $\Omega =1$ and 5 are portrayed in Fig. 4. It can be seen that the qualitative behavior of the numerical solutions is similar to that of the variational one. Namely, at $\alpha$ diminishing from 2 to its critical value $\alpha _{cr}(\Omega )$, the amplitude of the soliton grows so that at $\alpha \rightarrow \alpha _{cr}$ the solution resembles Dirac $\delta$ - function. To have better insight into the quantitative features of numerical and variational solutions, we plot the solutions for each $\alpha$ (except for $\alpha =1.1$ and lower) on separate panels. It is seen that while in the ”ordinary” case of $\alpha =2$ the numerical and variational solutions are similar to each other (this can also be considered as a ”sanity check” of our approach), at $\alpha <2$, the main difference of the variational and numerical solution occurs at the wings of the solution. Latter discrepancy becomes progressively larger as $\alpha$ approaches $\alpha _{cr}$. It is seen that at $\alpha =0.7$ at $\Omega =1$ and $\alpha =0.8$ at $\Omega =5$, the discrepancy on the wings is really large. We note also that as it was numerically difficult to obtain the solutions for $\alpha \rightarrow \alpha _{cr}$ corresponding to those in Fig. 1 (i.e. $\alpha =0.64$ for $\Omega =1$ and $\alpha =0.69$ for $\Omega =5$), we choose higher values $\alpha =0.7$ and 0.8 respectively, as shown on Fig. 4.

The main point behind those difficulties is the following. As our fractional soliton problem (8) formalizes as a boundary value problem for a nonlinear integral equation, its numerical solution is performed by the problem discretization and its reduction to the (very large to achieve reasonable accuracy) set of nonlinear algebraic equations (see, e.g.^34,35^), which then can be solved by (for instance) Newton-Raphson method. In our numerical calculations, to achieve satisfactory accuracy, we need to have around 10000 discretization steps on the interval (-20 - 20) (to have a ”reserve” for solution convergence as compared to the interval -5..5, which is depicted on Figures 1 and 4), which makes the task quite computer intensive. At the same time, as $\alpha \rightarrow \alpha _{cr}$, the above grid becomes insufficient to achieve desired accuracy so that we should choose a progressively larger number of steps, which requires an extremely long time to obtain (even not that accurate as for at higher $\alpha$) solution.

Our analysis shows that this outcome of variational treatment is since our simple trial function (8) has exponential asymptotics at infinities, while it is well-known (see, e.g.,^21–23,36^) that the asymptotics of the fractional differential equations solutions is usually power-law. Latter power-law asymptotics starts to manifest itself for $\alpha$’s somewhere close to $\alpha _{cr}$ for each dimensionless soliton frequency $\Omega =\omega /\omega _0$. To avoid this problem, we can construct more intricate trial function with asymptotics, dependent on Lévy index $\alpha$, see, e.g.^31–33,37^. Also, the use of a multi-parametric trial function would improve coincidence with numerics. Latter approaches, however, will complicate our analytical consideration a lot with no substantial gain in the understanding of the physical problem under consideration. This shows that our variational treatment with a simple one-parametric trial function captures well the soliton structure everywhere outside the narrow region near $\alpha _{cr}$. Below we will see that it gives also a very good approximation to the ”integral characteristics” of the soliton, like $N(\omega )$ as well as its phase diagram, i.e. lines $\alpha _{cr}(\Omega )$ and $dN/d\omega =0$. This shows that the considered variational approach could be considered as an efficient analytical tool to obtain and study the solution of the soliton equations with fractional derivatives.

**Figure 5:**
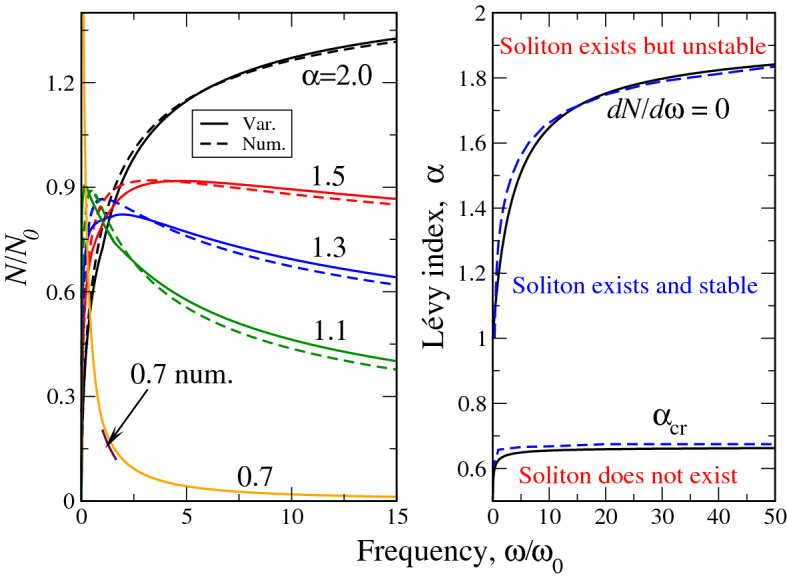
Comparison of numerical (dashed lines) and variational (solid lines) results for the soliton norm (left panel) and phase diagram (right panel). Due to numerical difficulties in the solution of the fractional equation (7) near the soliton existence boundary, only a small portion of the numerical curve $N/N_0(\Omega )$ (labeled ”0.7 num.”) is shown.

The comparison of analytical (variational) and numerical dependences $N(\omega )$ is reported in the left panel of Fig. 5. The numerical dependences $N(\omega )$ have been obtained by integration of the corresponding solutions at different $\alpha$. The quantitative coincidence with expression (23) is clearly seen: while at $\alpha =2$ the norm *N* grows everywhere, at $\alpha =1.5$ (essentially already at $\alpha <2$, see above), the maximum $N(\omega )$ appears, after which this function starts to decrease so that according to VK criterion, the soliton stabilizes. The little discrepancy in the positions of the $N(\omega )$ maxima is due to the discrepancy at the tails (or wings) of numerical and variational soliton solutions. It can be shown that for all $\alpha$ except those close to $\alpha _{cr}$, the maximal error between numerical and variational results is around 7%. As at $\alpha \rightarrow \alpha _{cr}$, we have difficulties (extremely long time of solution, see above) in obtaining the numerical solution of the equation (8), we opt to show only a small portion of the numerical curve $N/N_0(\Omega )$ on the left panel of Fig. 5.

Despite the above numerical difficulties of obtaining solutions near $\alpha _{cr}$, the curve $\alpha _{cr}(\Omega )$, signifying the soliton existence boundary can be calculated with sufficient accuracy. This curve along with stability boundary $dN/d\omega =0$ (which is not difficult to obtain numerically) is reported in the right panel of Fig. 5. The pretty good coincidence between numerical and variational curves is seen. The average error in this case is less than 5% so that out simple variational approach shows its reliability in calculation of the ”integral” soliton characteristics. The only thing we need now is to numerically check if really VK criterion of stability signifies it in the case of fractional textures. The crux of the matter here is that the VK criterion itself had been proven for ordinary (i.e. without fractional derivatives) soliton textures^18^ so that it is highly desirable to check the linear stability of our soliton solution by applying direct numerical simulations.

To accomplish the above task, we consider the linear stability problem for the soliton solution of the initial fractional NLSE (1) with ordinary Laplacian being substituted by fractional one (5). We look for its solution in the following complex form

24 $$\begin{aligned} \psi (x,t)=\left[ y(x)+p(x)e^{\lambda t}+q^*(x)e^{\lambda ^* t}\right] e^{i\omega t},\ p,q<<y. \end{aligned}$$

Here *y*(*x*) is unperturbed soliton texture (i.e. the above numerical solution of the equation (7)) and asterisk means complex conjugation. Substitution of (24) into fractional NLSE with its subsequent linearization over *p* and *q* generates following eigenvalue problem

25 $$\begin{aligned} {\hat{L}} p-f(x)q=i\lambda p,\ {\hat{L}} q-f(x)p=-i\lambda q, \nonumber \\ {\hat{L}}=-|\Delta |^{\alpha /2}+\omega -2gy^2(x)-3\chi y^4(x),\nonumber \\ f(x)=y^2(x)(g+2\chi y^2(x)), \end{aligned}$$

where $-|\Delta |^{\alpha /2}$ is defined by (4). By solving numerically the spectral problem (25), we obtain the spectrum of eigenvalues $\lambda =\lambda _r+i\lambda _i$, where $\lambda _{r,i}$ are, respectively, the real and imaginary parts. It can be shown that the necessary condition of *y*(*x*) stability is that all real parts $\lambda _r$ of the eigenvalues $\lambda$ are zero. In this case, the main role in the stability plays the sign of the lowest eigenvalue $i\lambda _i=p_0$. Namely, if $p_0<0$, the soliton is stable since the correction $\sim e^{\lambda t}=e^{-p_0t}$ to *y*(*x*) decays exponentially, i.e. after small perturbation, the system comes back to its initial state. In the opposite case $p_0>0$ we have exponentially growing perturbations and the soliton is unstable. Under lowest we understand the eigenvalue having largest modulus, in which case the exponential decay of the perturbation is strongest.

**Figure 6:**
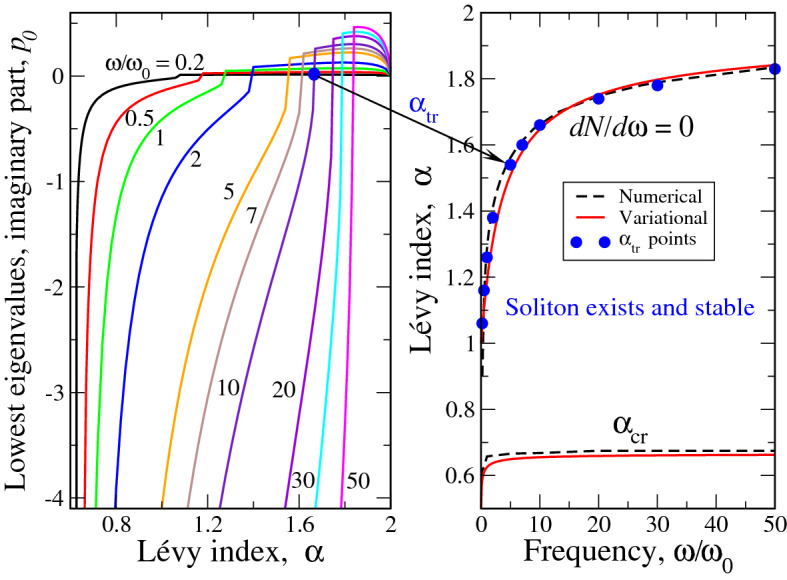
Left panel reports the imaginary part of the lowest eigenvalue $p_0$ as a function of Lévy index $\alpha$ at different $\Omega =\omega /\omega _0$ (figures near curves). For each $\Omega$, at $\alpha _{tr}<\alpha <2$, $p_0>0$ and the soliton is unstable. At the same time, at $\alpha _{cr}<\alpha <\alpha _{tr}$ $p_0<0$ and the soliton is stable. Arrow between left and right panels shows (specifically for $\Omega =10$, but this is also valid for all $\Omega$’s) that the points $\alpha _{tr}(\Omega )$ fall on the the curve $dN/d\omega =0$, defining the stability boundary according to VK criterion. The numerical and variational curves for stability ($dN/d\omega =0$) and soliton existence ($\alpha _{cr}(\Omega )$) boundaries in the right panel are shown as the guides for eye to delineate the range of stable solitons existence in our model.

The numerical behavior of $p_0$ versus $\alpha$ at different $\Omega$ (figures near curves) is reported in the left panel of Fig. 6. It is seen that for all $\Omega$ at $\alpha$ close to 2, the soliton is unstable as in this range $p_0>0$. The $p_0$ is positive until some threshold value $\alpha _{tr}$ of Lévy index, where $p_0$ changes its sign abruptly. Then, at $\alpha < \alpha _{tr}$, the sign of $p_0$ becomes negative, going to minus infinity almost vertically. This infinity is achieved at $\alpha =\alpha _{cr}$ (soliton existence boundary, see left panel of Fig. 3 and right panels of Figs. 5 and 6) so that at this point the soliton is ”infinitely stable” similar to the case of pure quintic nonlinearity^28^. We note in this context, that the case of pure quintic nonlinearity corresponds to $\omega _0 \rightarrow 0$ or $\Omega \rightarrow \infty$. Our calculation shows that as $\Omega$ becomes larger and larger than 50, the $\alpha _{tr} \rightarrow 2$ so that the range of positive $p_0$ collapses into a point $\alpha =2$ and soliton becomes stable as $\alpha$ become infinitesimally smaller than 2. This result is also in agreement with^28^.

If we take the points $\alpha _{cr}$ for each $\Omega$ from the left panel of Fig. 6 and plot them, we see immediately (blue points in the right panel of Fig. 6) that these points fall perfectly on the curve $dN/d\omega =0$, which defines the soliton stability boundary according to VK criterion. This means that our direct numerical simulations confirm the VK stability criterion for the ”fractional soliton” in a system with cubic-quintic nonlinearity. That being said, the soliton phase diagram in the right panel of Fig. 6 now becomes numerically corroborated. Note that the points $\alpha _{tr}$ coincide with the numerically (from the calculations of soliton textures, see Fig. 4) obtained stability boundary curve $dN/d\omega =0$ within 1% accuracy. The numerical curve, in turn, is very close to the variational one, see above. This once more shows that our simple variational approach, being much less laborious, gives very accurate information about the soliton structure and stability.

## Conclusions

It has been a principal goal of this article to investigate the structure and stability of the texture, emerging as a soliton solution of the fractional NLSE with cubic-quintic nonlinearity. The main outcome of present study is that to stabilize the initially unstable soliton texture, we do not need complex setups like optical lattice and/or traps for Bose-Einstein condensates. It turns out that to stabilize the soliton (although in our so far simple 1D model) it is sufficient to substitute the ordinary Laplacian in the NLSE to the fractional one. The possible rationale behind the latter substitution is that the ”fractionalization” (say, the deviation od Lévy index $\alpha$ form 2 in a fractional Laplacian) may come from the strong (i.e. non-Gaussian) disorder in a system^31–33^.

The major argument here is that a disordered system typically has a wide, non-Gaussian distribution of its characteristics^33^. Such strong disorder in quantum mechanics typically causes the localization of initially (before the disordering of a sample like its amorphization) itinerant states. The renowned Anderson localization^38^ can be explained in this way. This refers to ”greater localization” of the soliton in the context of our problem, such as its collapse at $\alpha =\alpha _{cr}$ (Figs. 1 and 4), so that it becomes ”more stable” than in the ordinary ”non-fractional” situation, which corresponds to $\alpha =2$.

Here, using analytical (variational) and numerical arguments, we construct the soliton phase diagram (ranges of its existence and stability) in terms of variables $\Omega =\omega /\omega _0$ (or, concurrently $\lambda$, see Eq. (8)) - $\alpha$. This phase diagram shows that soliton exists and is stable in the wide range of its nonlinearity coefficients *g* and $\chi$ (which merge in a single parameter $\Omega$ or $\lambda$) as well as disorder strength, characterized by the Lévy index $\alpha$. Moreover, Fig. 6 shows that the soliton stability boundary, obtained from VK criterion^18^ coincides with that, obtained by direct numerical solution of linear stability problem (25). This means that for our system with mixed cubic-quintic nonlinearity, the VK criterion gives a reliable answer on the soliton stability question. This, in turn, signifies that our simple variational approach with two-parameters trial function, being much less laborious, then direct numerical solution of nonlinear integral equation (7), gives very accurate (within few per cent accuracy) information about soliton structure and stability. Our preliminary results show that the same variational method can be well applied to the systems with more complex nonlinearity (like saturation one) as well as to systems in higher dimensions. We note, that for 2D and 3D systems, the numerical solution of corresponding fractional equation becomes prohibitively slow even in the problems with cylindrical (2D) or spherical (3D) symmetries. This is because the 2D or 3D interval $0<r<\infty$ (rather than $-\infty<x<\infty$ in our 1D case) imposes additional limitations on the iterative solutions of the set of nonlinear equations, obtained from the initial integral one. The results of these studies will be published elsewhere.

We note that here, similar to the case of purely quintic nonlinearity^28^, we can develop the perturbation theory with the small parameter being the deviation of $\alpha$ from 2. The point is that such perturbational expansion is the only analytical instrument available to study the soliton solutions in models of any complexity when variational solution is impossible for some reasons. Although perturbational calculations (particularly in higher orders and for models with complex nonlinearities, notably in the external potentials) can be very cumbersome, they serve as guidance for numerical simulations as the nonlocal nature of fractional derivatives makes even the numerical computations challenging. Latter is especially true for higher spatial dimensions. Also, the stability effects (for example using VK criterion, which should then be checked numerically) can be studied analytically within the latter technique. On the other hand, the perturbational solution can be viewed as ”exact” in the range of its applicability. As a result, it is possible to think of a perturbational soliton as complementary to a variational one near $\alpha =2$. This is largely true for the models (including our with mixed cubic-quintic nonlinearities), where we have the exact solutions for the ”ordinary” case $\alpha =2$. Latter solution gives extremely convenient zeroth approach to further develop the perturbation theory.

The results reported here may be used to control (e.g. by varying Lévy index $\alpha$ and/or nonlinearity coefficients) the properties of nonlinear systems with a significant disorder. To be specific, it is common knowledge that disorder (especially strong like crystalline solid amorphization) is usually considered as an unwanted effect. However, in recent years, its helpful features have grown more and more apparent. Specifically, the impacts of disorder provide an extra opportunity to fine-tune the system’s physical properties in addition to standard external stimuli like electromagnetic fields, optical lattices, and/or confining potentials. Specifically, by manipulating the type and concentration of different imperfections (like point or extended defects in solids used in nonlinear optics), we can alter the physical properties of a disordered host in the desired direction. In other words, this enables us to modify a system’s features to satisfy specific requirements, needed, for instance, in optoelectronics. It had been demonstrated in recent papers^6,10^ that there are many physical systems, where the balance between nonlinearity and fractional, disorder-driven, dispersion, produces many intriguing effects, which do not occur in ordinary (i.e. those with Lévy index $\alpha =2$) systems. This is also true in systems with Bose-Einstein condensates having fractional dispersion^11^.

## Methods

The details of our theoretical methodology and those of working with fractional derivatives and fractional Laplacians, in particular, have been described in the sections “The model” and ”Analytical results: the variational approach”. The numerical solutions of boundary value problems for fractional NLSE have been conducted using the commercial *Mathematica* software package as well as C++ routines, partially written *ad hoc* and partially taken from the standard libraries like LAPACK.

## Supplementary Information

Supplementary Information.

## Data Availability

The datasets used and/or analysed during the current study available from the corresponding author on reasonable request.
