# Supplementary material for: 1D solitons in cubic-quintic fractional nonlinear Schrödinger model
Source: Sci Rep. 2022 Sep 2;12:15031. doi: 10.1038/s41598-022-19332-z (PMC9440054; doi:10.1038/s41598-022-19332-z)
Supplement: Supplementary file 1 — Supplementary Information. [file 41598_2022_19332_MOESM1_ESM.pdf]

Supplementary material to the article "1D solitons in cubic-quintic fractional nonlinear Schrödinger model"

V. A. Stephanovich,[1,*] W. Olchawa,[1] E.V. Kirichenko,[1] V.K. Dugaev[2]

[1] Institute of Physics, University of Opole
ul. Oleska 48, 45-052 Opole, Poland
[2] Department of Physics and Medical Engineering,
Rzeszów University of Technology,
al. Powstańców Warszawy 6, 35-959 Rzeszów, Poland

August 17, 2022

1 Appendix

To derive the expression for fractional Laplacian (5), one can start either from the famous Lévy - Khintchine theorem [20, 29] or from Riemann–Liouville fractional integral [39], see also Appendix A of Ref. [30] and references therein. Concisely, the Riesz fractional derivative (fractional Laplacian in physical problems) is the symmetrization of the corresponding Riemann-Liouville integrals [29, 30]. In the 1D case, this reduces to the sum of corresponding integrals in the negative and positive real semiaxis [30]. However, to get more insights into the properties of the Fourier image of the fractional Laplacian, it is more profitable to begin with the Lévy - Khintchine theorem (formula), which deals with the characteristic function (Fourier image) of the so-called infinitely divisible probability distributions. Latter comprise both Lévy (distributions that describe the system having long jumps, [20, 29]) and Gaussian distributions. The other advantage of the Lévy-Khintchine formula is that in coordinate space, it gives already the symmetric, Riesz type of fractional derivative [20, 29, 39]. In a one-dimensional setting, the part of the Lévy-Khintchine formula, related to the non-Gaussian, jump-type stochastic processes, reads (see Refs [20, 29] for details)

$$|\Delta|^{\alpha/2}g(x)=\int_{-\infty}^{\infty}\left[g(x+y)-g(x)-\frac{yf'(x)}{1+y^2}\right]\nu_\alpha(dy), \tag{1}$$

where $f'(x) = df/dx$ and $\nu_\alpha(dy)$ stands for Lévy measure with index α. In our 1D case

$$\nu_\alpha(dy) = A_\alpha \frac{dy}{|y|^{1+\alpha}}. \tag{2}$$

It is seen that at $y = 0$, the expression (1) is divergent. This means that the integral (1) exists only in the sense of its Cauchy principal value. Really, the expansion of the integrand (1) at small y yields $y^3 f'(x)/(1+y^2) \approx y^3 f'(x)$, which is an odd function. As the Lévy measure (2) contains the even function of y, the integral gives zero in the close vicinity of $y = 0$

$$\lim_{\varepsilon \to 0} \int_{-\varepsilon}^{\varepsilon} \frac{y^3 dy}{|y|^{1+\alpha}} = 0. \tag{3}$$

This feature makes the numerical calculation of the integrals like (1) very troublesome. That is why, in many physical problems, it is more profitable not to consider the integral (1) directly, but to pass to its Fourier representation.

To be able to calculate the Fourier image of a fractional Laplacian in the closed form, in a majority of physical problems, it is customary to omit the third term in the integrand (1), considering the integral (cf. (5))

$$|\Delta|^{\alpha/2} g(x) = A_\alpha \int_{-\infty}^{\infty} \Big[g(x+y) - g(x) \Big] \frac{dy}{|y|^{1+\alpha}}. \tag{4}$$

This integral also exists in the sense of its Cauchy principal value. The corresponding integrand near $y = 0$ expands as $yf'(x)$ so that the integral like (3) also gives zero.

To calculate the Fourier image of (4), we define the Fourier pair

$$g(k) = \int_{-\infty}^{\infty} g(x) e^{ikx} dx, \;\; g(x) = \frac{1}{2\pi} \int_{-\infty}^{\infty} g(k) e^{-ikx} dk \tag{5}$$

and substitute the second expression (5) into (4). We obtain

$$|\Delta|^{\alpha/2} g(x) = \frac{A_\alpha}{2\pi} \int_{-\infty}^{\infty} \frac{dy}{|y|^{1+\alpha}} \left[\int_{-\infty}^{\infty} g(k) e^{-ik(x+y)} dk - \int_{-\infty}^{\infty} g(k) e^{-ikx} dk \right] =$$
$$= \frac{A_\alpha}{2\pi} \int_{-\infty}^{\infty} g(k) e^{-ikx} dk \int_{-\infty}^{\infty} \frac{(e^{-iky} - 1) dy}{|y|^{1+\alpha}}. \tag{6}$$

The integral over dy in (6) exists in the sense of its Cauchy principal value only. It can be calculated explicitly to give

$$\int_{-\infty}^{\infty} \frac{(e^{-iky} - 1) dy}{|y|^{1+\alpha}} = 2|k|^\alpha \Gamma(-\alpha) \cos \frac{\pi\alpha}{2}. \tag{7}$$

In other words, the Fourier image of the "almost divergent" integral operator (4) is continuous (everywhere except the point $k = 0$) as its "most divergent" part is "hidden" in the integral (7), which can be calculated explicitly. It is seen

from (7) that at the limiting value $\alpha = 0$, $\Gamma(0)$ is divergent so that the integral (7) is divergent as well. The same happens for $\alpha = 2$, in view of the divergence of $\Gamma(-2)$. However, it can be shown, that irrespective of how close to 0 or 2 the Lévy index α is, the integral (7) is convergent. This is the source of strict inequality $0 < \alpha < 2$, where the fractional Laplacian exists.

The substitution of (7) into (6) yields

$$|\Delta|^{\alpha/2} g(x) = \frac{2A_\alpha \Gamma(-\alpha)\cos\frac{\pi\alpha}{2}}{2\pi} \int_{-\infty}^{\infty} |k|^\alpha g(k) e^{-ikx} dk =$$
$$= \frac{1}{2\pi} 2\sin\frac{\pi\alpha}{2} \cos\frac{\pi\alpha}{2} \frac{\Gamma(-\alpha)\Gamma(1+\alpha)}{\pi} \int_{-\infty}^{\infty} |k|^\alpha g(k) e^{-ikx} dk \equiv$$
$$\equiv -\frac{1}{2\pi} \int_{-\infty}^{\infty} |k|^\alpha g(k) e^{-ikx} dk, \tag{8}$$

which is exactly the expression (6) from the main text. Here we use the identity

$$\Gamma(1+\mu)\Gamma(-\mu) = -\frac{\pi}{\sin \pi\mu}. \tag{9}$$

It is seen from (8), that at $\alpha = 2$, the fractional Laplacian converts to the ordinary second spatial derivative.

References

[1] Y. S. Kivshar, G. P. Agrawal, *Optical Solitons: From Fibers to Photonic Crystals* (Academic, San Diego, California 2003).

[2] C. Sulem and P. L. Sulem, *The nonlinear Schrödinger Equation: Self-Focusing and Wave Collapse.* (Springer, Berlin, 1999).

[3] *Emergent Nonlinear Phenomena in Bose-Einstein Condensates* Editors: P. G. Kevrekidis, D. J. Frantzeskakis and R. Carretero-González, (Springer-Verlag, Berlin, 2008).

[4] Y. V. Kartashov, G. E. Astrakharchik, B. A. Malomed, L. Torner, Frontiers in multidimensional self-trapping of nonlinear fields and matter, Nat. Rev. Phys. **1**, 185 (2019).

[5] Y. V. Kartashov, B. A. Malomed, L. Torner, Solitons in nonlinear lattices, Rev. Mod. Phys. **83**, 247 (2011).

[6] D. Mihalache, Localized structures in optical and matter-wave media: a selection of recent studies, Rom. Rep. Phys. **73**, 403 (2021).

[7] F. Kh. Abdullaev & M. Salerno, Gap-Townes solitons and localized excitations in low-dimensional Bose-Einstein condensates in optical lattices, Phys. Rev. A **72**, 033617 (2005).

[8] Yu. B. Gaididei, J. Schjødt-Eriksen, and P. L. Christiansen, Collapse arresting in an inhomogeneous quintic nonlinear Schrödinger model, Phys. Rev. E **60**, 4877 (1999).

[9] N. N. Akhmediev and A. Ankiewicz, *Solitons: Nonlinear Pulses and Beams* (Kluwer, Boston, 1997).

[10] B. A. Malomed, Optical solitons and vortices in fractional media: a mini-review of recent results, Photonics **8**, 353 (2021).

[11] H. Sakaguchi and B.A. Malomed, One- and two-dimensional solitons in spin–orbit- coupled Bose–Einstein condensates with fractional kinetic energy, Journal of Physics B, **55**, 155301 (2022).

[12] F. Kh. Abdullaev, A. Gammal, L. Tomio, and T. Frederico, Stability of trapped Bose-Einstein condensates, Phys. Rev. A **63**, 043604 (2001).

[13] W. Zhang, E. M. Wright, H. Pu, and P. Meystre, Fundamental limit for integrated atom optics with Bose-Einstein condensates, Phys. Rev. A **68**, 023605 (2003).

[14] A. Biswas and S. Konar, *Introduction to non-Kerr law optical solitons* (CRC Press, New York, 2006).

[15] Kh. I. Pushkarov, D. I. Pushkarov and I. V. Tomov, Self-action of light beams in nonlinear media: soliton solution, Opt. Quant. El., **11**, 471 (1979).

[16] D. Pathria and J. L. Morris, Exact solutions for a generalized nonlinear Schrodinger equation, Phys. Scr., **39**, 673 (1989).

[17] D. E. Pelinvosky, Y. S. Kivshar and V. V. Afanasjev, Instability-induced dynamics of dark solitons, Phys. Rev. E **54**, 2015 (1996).

[18] N.G. Vakhitov and A.A. Kolokolov, Stationary solutions of the wave equation in a medium with nonlinearity saturation, Izv. Vysshikh Uchebnych Zavedenii, Radiofizika, **16**, 1020, 1973.

[19] F. Kh. Abdullaev and J. Garnier, Dynamical stabilization of solitons in cubic-quintic nonlinear Schrödinger model, Phys. Rev. E **72**, 035603(R) (2005).

[20] P. Lévy, *Théorie de l'addition des variables aléatoires* (Gauthier-Villars, Paris, 1954).

[21] *Lévy Flights and Related Topics in Physics*, edited by M. F. Shlesinger, G. M. Zaslavsky, and U. Frisch, Lecture Notes in Physics (Springer-Verlag, Berlin, 1995).

[22] R. Metzler and J. Klafter, The restaurant at the end of the random walk: recent developments in the description of anomalous transport by fractional dynamics, J. Phys. A: Math. Gen. **37**, R161 (2004).

[23] A. A. Dubkov, B. Spagnolo, and V. V. Uchaikin, Lévy flight superdiffusion: an introduction, Int. J. Bifurcat. Chaos **18**, 2649 (2008).

[24] N. Laskin, *Fractional Quantum Mechanics* (World Scientific, Singapore, 2018).

[25] R.P. Feynman and A. R. Hibbs *IQuantam mechanics and path integrals* (Dower, New York, 2010).

[26] P. Li, B. A. Malomed and D. Mihalache, Vortex solitons in fractional nonlinear Schrödinger equation with the cubic-quintic nonlinearity, Chaos, Solitons and Fractals **137**, 109783 (2020).

[27] L. Zeng, D. Mihalache, B. A. Malomed, X. Lu, Yi Cai, Q. Zhu and J.Li, Families of fundamental and multipole solitons in a cubic-quintic nonlinear lattice in fractional dimension, Chaos, Solitons and Fractals **144**, 110589 (2021).

[28] V.A. Stephanovich and W. Olchawa, Stabilization of 1D solitons by fractional derivatives in systems with quintic nonlinearity, Scientific Reports, **12**, 384 (2022). https://doi.org/10.1038/s41598-021-04292-7.

[29] D. Applebaum, *Lévy Processes and Stochastic Calculus.* (Cambridge University Press, Cambridge, 2009).

[30] D. Brockmann and I.M. Sokolov, Lévy flights in external force fields: from models to equations, Chemical Physics **284**, 409 (2002).

[31] E. V. Kirichenko and V.A. Stephanovich, The influence of disorder on the exciton spectra in two-dimensional structures, PCCP **21**, 21847 (2019).

[32] V.A. Stephanovich and W. Olchawa Lévy distributions and disorder in excitonic spectra, PCCP, **22**, 24462 (2020).

[33] E. V. Kirichenko and V.A. Stephanovich, The influence of Coulomb interaction screening on the excitons in disordered two-dimensional insulators, Scientific Reports, **11**:11956 (2021). https://doi.org/10.1038/s41598-021-91414-w.

[34] A.D. Polanin and A.V. Manzhirov *Handbook of Integral Equations.* Second Edition. (Taylor and Francis, Boca Raton, 2008).

[35] W.H. Press, S.A. Teukolsky, W.T. Vetterling and B.P. Flannery *Numerical Recipes: The Art of Scientific Computing.* Third Edition. (Cambridge University Press, Cambridge, 2007)

[36] S. G. Samko, A. A. Kilbas, and O. I. Maritchev, *Fractional Integrals and Derivatives.* (Gordon and Breach, New York, 2003).

[37] E. V. Kirichenko and V.A. Stephanovich, Confinement of Lévy flights in a parabolic potential and fractional quantum oscillator, Phys. Rev. E **93**, 052110 (2018).

[38] P.W. Anderson, Absence of diffusion in certain random lattices. Phys. Rev. **109**, 1492 (1958).

[39] M. Riesz, L'intégrale de Riemann–Liouville et le problème de Cauchy. Acta Math. **81** 1 (1949).
